# Prevalence of asthma, rhinitis and eczema symptoms in rural and urban school-aged children from Oropeza Province - Bolivia: a cross-sectional study

**DOI:** 10.1186/1471-2466-14-40

**Published:** 2014-03-10

**Authors:** María Teresa Solis Soto, Armando Patiño, Dennis Nowak, Katja Radon

**Affiliations:** 1 Center for International Health, Ludwig-Maximilians-University, Ziemssenstr. 1, 80336 Munich, Germany; 2 Departmental Service of Health (SEDES) – Chuquisaca, 202 Rosendo Villa Street, Sucre, Bolivia; 3 Institute for Occupational, Social and Environmental Medicine, Occupational and Environmental Epidemiology & Net Teaching Unit, University Hospital Munich (LMU), Munich. Ziemssenstr. 1, 80336 Munich, Germany

**Keywords:** Asthma, Rhinoconjunctivitis, Eczema, Children, Prevalence, Bolivia

## Abstract

**Background:**

Asthma and allergies are world-wide common chronic diseases among children and young people. Little information is available about the prevalence of these diseases in rural areas of Latin America. This study assesses the prevalence of symptoms of asthma and allergies among children in urban and rural areas at Oropeza Province in Bolivia.

**Methods:**

The Spanish version of the ISAAC standardized questionnaire and the ISAAC video questionnaire were implemented to 2584 children attending the fifth elementary grade in 36 schools in Oropeza province (response 91%). Lifetime, 12 months and severity prevalence were determined for asthma, rhinitis and eczema symptoms. Odds ratios (OR) with 95% confidence intervals (95% CI) were calculated adjusting for age using generalized linear mixed-effects models.

**Results:**

Median age of children was 11 years, 74.8% attended public schools, and 52.1% were female. While children attending urban schools had lower prevalence of self-reported wheeze in the written questionnaire (adjusted OR 0.6; 95% CI 0.4-1.9), they were more likely than children attending rural schools to report wheeze in the video questionnaire (aOR 2.1; 95% CI 1.0-2.6). They also reported more frequently severe rhinoconjunctivitis (aOR 2.8; 95% CI 1.2-6.6) and severe eczema symptoms (aOR 3.3; 95% CI 1.0-11.0).

**Conclusion:**

Overall in accordance with the hygiene hypothesis, children living in urban areas of Bolivia seem to have a higher prevalence of symptoms of asthma and allergies compared to children living in the country side. In order to develop primary prevention strategies, environmental factors need to be identified in future studies.

## Background

Asthma and allergies are world-wide common chronic diseases with high prevalences among children and young people and large variation between and within countries and cities [1]. These pathologies are associated with adverse outcomes at physical, emotional, social, and professional level for both, patients and their families, interfering with normal activity and quality of life [2].

Among others, the International Study of Asthma and Allergies in Childhood (ISAAC) has shown a higher prevalence of symptoms of asthma and allergies in industrialized countries than in developing countries [3-5], becoming a new public health challenge for the region [6].

ISAAC Phase I reported that the prevalence of asthma symptoms in children from South America was as high as that reported from industrialized countries. Overall, the prevalence of recent wheeze, exercise-induced bronchospasm, and severe episodes of wheezing were 16.9%, 19.3%, and 4.6%, respectively, for children aged 13–14 years with marked differences in the prevalence of asthma symptoms between different countries [7]. In ISAAC Phase III, after a period of 5–10 years, similar or even lower prevalences of asthma symptoms were reported in centers with high prevalence in ISAAC Phase I. This suggests that the prevalence of asthma has reached a plateau in high prevalence centers while it is still on the rise in low and intermediate prevalence centers. The same scenario was reported for rhinoconjunctivitis and eczema symptoms [8].

Changes in lifestyle, modernization, living conditions and dietary habits have been studied as possible explanations for such an increase [9]. One aspect frequently shown for industrialized countries is a higher prevalence of symptoms of asthma and allergies in urban compared to rural areas. This association was confirmed for African and Asian countries [10,11] but so far only few studies from Latin America exist [12,13]. A higher exposure to microbial compounds in rural areas was discussed as one of the mechanisms underlying the lower prevalence of asthma and allergies in rural areas. However, a high prevalence of asthma was shown in inner city environments with high microbial load [14,15]. Some authors have speculated that these contradictory findings might be related to different asthma phenotypes and have hypothesized that rural living protects from allergy but not from asthma [16-18]. However, recent studies do not confirm this hypothesis [19,20].

Bolivia is the poorest country in the Latin American region with 31% of the population living in extreme poverty [21]. Living conditions differ largely between urban and rural areas [22]. Within the ISAAC study, the prevalence of asthma and allergies in Bolivia has only been assessed for Santa Cruz, Bolivia’s richest city [23]. The study has shown a high prevalence of rhinoconjunctivitis [4,6] and eczema symptoms [24] as compared to other Latin American countries but a moderate prevalence of asthma [6]. So far, data on the prevalence of asthma and allergies neither exist from lower income urban areas nor from rural areas of Bolivia.

This study aimed to assess the prevalence of symptoms of asthma and allergies in urban and rural areas at Oropeza Province in Chuquisaca. In this region, the majority of people live in poverty (62%), and 66% of the population identify themselves with some ethnic group (Aymara, Quechua, Guaraní, among others) [25]. By assessing the burden of disease this study will contribute to assess the necessity to allocate recourses to the treatment, primary and secondary prevention of asthma and allergies in a country where health resources are scarce.

## Methods

### Design

A population-based prevalence study was conducted in the Oropeza Province, located in the north western part of Chuquisaca, from July 2011 to December 2011.

### Setting

The estimated population of Oropeza province is around 336.218 inhabitants with 19.7% living in the rural area. Communities with less than 2000 inhabitants were considered rural [26].

Oropeza’s population lives at altitudes between 2000 and 4000 meters above sea level. Its climate is warm and dry, with temperatures between 15.0°C and 20.0°C, relative humidity mean annual value around 60%. Rainfall occurs predominantly in summer (average annual rainfall of 500 to 1000 cubic millimeters) [27].

### Study population and field work

This study used a simple one-stage cluster sampling. The primary sampling units were schools selected randomly. From a total 185 school registered in the Regional Education Service (SEDUCA- Chuquisaca) (138 (75%) in urban areas and 47 (25%) in rural areas), 43 schools were selected randomly (36 (84%) in urban, 7 (16%) in rural areas). Of these, 6 urban (17%) and 1 rural school (14%) did not accept to participate due to time constraints or because of parental concern.

The secondary sampling units were all students in fifth grade. From the 36 participating schools (30 (83%) of these located in urban areas), 2584 children attending the fifth grade were invited to participate. In each sampled school, the whole fifth grade student population was studied. Schools with less than 20 pupils of this grade were excluded from the sampling frame for feasibility reasons.

The sample size was determined following ISAAC project recommendations [28]. With 2016 participants is possible to detect differences in the prevalence of severe asthma considering 5% of significance level and 90% of statistical power.

The schools were contacted to agree upon the date of the visit, and to organize the written and video questionnaire ensuring a place to implement both questionnaires properly.

After the written questionnaire, the ISAAC video questionnaire (AVQ 3.0) was implemented according the ISAAC standard recommendations [29]. The main researcher (MTSS) and three previously trained health professionals submitted the questionnaires.

### Study instruments and variable definition

#### *Written and video questionnaires*

The Spanish version of the ISAAC standardized questionnaire (asthma, rhinitis and eczema modules) was used [30]. This questionnaire attempts to identify children with symptoms of asthma, rhinoconjunctivitis and eczema. The written questionnaire assesses lifetime (ever) and 12 months prevalence (current symptoms) of asthma, rhinitis and eczema symptoms. In addition, severity of symptoms is assessed. In addition, the video questionnaire identifies 12 months prevalence of asthma symptoms.

The following definitions based on the ISAAC protocol were used:

*Asthma symptoms:* were defined as self-reported symptoms of wheezing or whistling in the chest.

*Severe asthma symptoms:* was considered present if children with current asthma symptoms reported: 4 or more attacks of wheeze, being woken by wheeze at one or more nights per week or wheezing severe enough to limit speech to only one or two words at a time between two breaths.

Considering the video questionnaire, *wheezing, exercise-induced wheeze and dyspnea at rest* occurrence were considered by affirmative response to the respective video sequences.

*Rhinitis symptoms* were defined as self-reported problems of sneezing, runny or blocked nose, without having a cold or influenza.

*Rhino conjunctivitis symptoms* were considered present if participants reported rhinitis symptoms accompanied by itchy watery eyes.

*Severe allergic rhinitis symptoms* was defined as rhinoconjunctivitis symptoms interfering with daily activities [4,28].

*Itchy Rash* was considered if the participant reported an itchy rash which was coming and going for at least six months.

*Current symptoms of eczema* were defined as presence of an itchy rash in the last 12 months that affected any of the following places: the folds of the elbows, behind the knees, in front of the ankles, under the buttocks, or around the neck, ears or eyes.

*Severe eczema symptoms* was considered present if eczema symptoms affected sleep (1 or more nights per week) [5,28].

### Statistical analysis

Double-entry of data and congruence checking was performed using the statistical analysis software EpiInfo V. 3.5.3 for Windows to avoid possible mistakes. Data was exported to SPSS v.17 for descriptive analysis and to R v. 3.0.1 [31] software for statistical models.

Intraclass correlation coefficient (ICC) was computed [32] to analyze the relationship of cases within a cluster (schools) for each of the outcomes. ICC is around 4% for outcomes reported by written questionnaire and around 20% for outcomes reported by Video questionnaire. Considering that, all analyzes were adjusted by cluster sampling.

Absolute and relative frequencies were calculated. The Rao-Scott adjusted chi-square test [33] was computed to assess differences in prevalence of symptoms between rural and urban areas. In addition crude and adjusted odds ratios as well as their corresponding 95% confidence intervals were calculated using generalized linear mixed-effects models [34]. The adjusted models included variables which were statistically significant (p value ≤ 0.05) in the bivariate analysis.

### Ethics

The study was approved by the National Research Ethics Committee from San Andres University at La Paz–Bolivia; also a permission to apply the questionnaire was obtained from the Regional Education Service (SEDUCA- Chuquisaca). International ethical research guidelines were considered at all research steps. A written informed consent form as well as a letter explaining the importance of the study was sent to parents or legal guardians of the children one week before the visit to the school. Voluntary participation from children was respected.

## Results

In the participating schools, response was 91%. The median age of participating students was 11 years (Range from 9 to 15 years) with children from urban schools being younger and more likely to attend public schools than children from rural schools (Table 1).

**Table 1:** Descriptive data by for place of living (N = 2340)

		**Urban**		**Rural**		p value*
		**(N = 1727)**		**(N = 613)**		
		**n**	**%**	**n**	**%**	
**Sex**	Female	917	53.2	300	48.9	0.14
**Age**	≤ 10 years	822	48.0	175	28.6	< 0.01
	11 years	672	39.3	303	49.4	
	≥ 12 years	218	12.7	135	22.0	
**Type of school**	Public	1400	81.1	351	57.3	0.17
	Private and public with private Infrastructure	327	18.9	262	42.7	

*Chi square test.

### Asthma symptoms

Based on the written questionnaire, the 12-months prevalence of asthma symptoms was 16.4% in urban vs. 21.7% in rural areas while in the video questionnaire asthma symptoms were reported by only 7.3% of urban and 3.9% of rural participants (Table 2). Adjusting for age, sex and type of school these differences were confirmed (urban vs. rural schools: written questionnaire OR 0.6; 95% CI 0.4-1.9; video questionnaire OR 2.1; 95% CI 1.0-2.6).

**Table 2:** Prevalence, unadjusted and adjusted odds ratios comparing self-reported asthma, rhinitis and eczema in school aged children from rural and urban areas

	**Urban**		**Rural**		**Crude OR**	**Adj. OR**
	**n**	**%**	**n**	**%**	**95% CI**^**b**^	**95% CI**^**b,c**^
**ASTHMA SYMPTOMS**						
Written questionnaire (N = 2340)						
Wheeze (ever)	470	27.4	214	34.9	0.7 (0.4-1.9)	0.7 (0.4-1.9)
Wheeze (12 months prevalence)	281	16.4	133	21.7	0.6 (0.4-1.9)	0.6 (0.4-1.9)
Severe asthma^a^ (12 months prevalence)	158	9.2	76	12.4	0.7 (0.4-1.9)	0.7 (0.5-1.9)
Video questionnaire (N = 2300)						
Wheezing (12 months prevalence)	124	7.3	24	3.9	2.3 (1.1-2.7)	2.1 (1.0-2.6)
Exercise-induced wheeze (12 months prevalence)	323	19.2	80	13.1	1.7 (0.9-2.4)	1.7 (0.9-2.4)
Dyspnoea at rest (12 months prevalence)	68	4	17	2.8	1.8 (0.7-3.1)	1.7 (0.7-3.2)
**RHINITIS SYMPTOMS** (N = 2327)						
Rhinitis (ever)	715	41.7	255	41.6	1.0 (0.7-1.6)	1.0 (0.7-1.6)
Rhinitis (12 months prevalence)	490	28.6	175	28.5	1.0 (0.7-1.6)	1.0 (0.8-1.6)
Rhinoconjunctivitis^d^ (12 months prevalence)	369	21.5	147	24.0	0.8 (0.6-1.8)	0.8 (0.6-1.8)
Severe Rhinoconjunctivitis^e^ (12 months prevalence)	43	2.5	6	1.0	2.6 (1.1-7.8)	2.8 (1.2-6.6)
**ECZEMA SYMPTOMS** (N = 2303)						
Itchy rash (ever)	339	20	123	20.1	1.0 (0.7-1.7)	1.0 (0.7-1.7)
Itchy rash (12 months prevalence)	203	12.0	56	9.2	1.4 (0.9-1.8)	1.4 (1.0-1.8)
Eczema^f^ (12 months prevalence)	161	9.5	52	8.5	1.1 (0.7-1.8)	1.2 (0.8-1.8)
Severe Eczema^g^ (12 months prevalence)	27	1.6	3	0.5	3.3 (1.0-10.9)	3.3 (1.0-11.0)

^a^ ≥ 4 attacks of wheeze or woken by wheeze on one or more nights per week or wheezing severe enough to limit speech to only one or two words at a time, between breaths.

^b^Reference category: rural.

^c^Adjusted by age.

^d^Presence of sneezing or a runny or blocked nose, accompanied by itchy watery eyes without a cold or the flu.

^e^Rhinoconjunctivitis symptoms interfering with daily activities (a lot).

^f^Presence of an itchy rash at any time in the past 12 months affecting the following places: the folds of the elbows; behind the knees; in front of the ankles; under the buttocks; or around the neck, ears, or eyes).

^g^Sleep disturbed by rash in the last 12 months in children with eczema symptoms (one or more nights per week).

### Rhinitis symptoms

The prevalence of rhinitis symptoms was high with a 12-months prevalence of rhinoconjunctivitis symptoms of 22% in children from urban and 24% in children from rural schools. Only for severe rhinoconjunctivitis symptoms the 12-months prevalence differed between urban and rural schools (aOR 2.8; 95% CI 1.2-6.6; Table 2).

### Eczema symptoms

The 12-months prevalence of current itchy rash was higher in urban than in rural schools (12% vs. 9%; Table 2). The 12-months prevalence of severe eczema was very low (1.6% in children from urban schools, 0.5% in children from rural schools). Only the 12-months severe eczema symptoms was statistically significantly higher in children from urban compared with children from rural schools (aOR 3.3; 95% CI 1.0-11.0).

## Discussion

Our study for the first time assessed the prevalence of symptoms of asthma and allergies in rural and urban areas of Bolivia. The prevalence found in this study was within the range reported for South America [4,6,24]. Difference between rural and urban areas indicated higher prevalences for urban areas compatible with the hygiene hypothesis, although the difference was smaller in comparison with previous studies [15].

This is the first study in Bolivia comparing the prevalence of symptoms of asthma and allergies in a representative sample of children in this region using standardized methods. The study showed an optimal response rate (91%), which is similar to the one reported from other studies following the ISAAC protocol in Latin America [6].

However, 7 out of the selected 43 schools did not agree to participate (16%). Most of these schools were from urban areas. The main reason for non-participation was the frequent implementation of different governmental programs which interrupt the education schedule. It is not assumed that this might have resulted in selection bias.

Although cluster effect was not expected to be large in ISAAC studies [28], methods for correlated data were computed as recommended when cluster sampling is implemented [35]. In our study, even if we found an ICC of 20% in some cases, huge variations between logistic regression models (considering independent data) and generalized linear mixed-effects models (considering the correlation between schools) were not founded.

In contrast to the original ISAAC protocol, we included children in the 5^th^ grade (age range 9–15 years). The reason for that was that in the following school grades the drop-out rate in Bolivian schools is substantial most likely affecting the representativeness of the results [36]. We considered that these children are able to understand and adequately respond the questionnaire. The alternative to select 1^st^ grade pupils and use the International Study of Asthma and Allergies in Childhood parental questionnaire was omitted due the overall low literacy level among adults in this region.

Another limitation of our study is related to the cross-sectional design, with the exposure and outcome being measured simultaneously, thus it is not possible to determine the temporality of the association or the causality. Although this design is prone to induce recall bias, we assume that blinding the participants to the hypothesis under investigation minimized this concern.

In accordance with previous studies, we have found a low agreement between written and video questionnaire for asthma with much higher prevalences in the written than in the video questionnaire [37]. Given the extremely high prevalence in the written asthma questionnaire as compared to the video questionnaire and to other studies in Latin America [8] one might assume that the results reported by video questionnaire could be more reliable than those from written questionnaire.

The video questionnaire has been described as being sensitive to language, culture, or literacy differences [37,38]. Even given that the video questionnaire has been validated only in children 13 to 14 year olds, it is unlikely that its validity decreases so much with age (median age of our population: 11 years). Even if this would be the case it is expected to result in non-differential bias which might lower the risk estimates towards the null.

Overall, the prevalence of current asthma symptoms (6.4%) were lower than the Latin American average (10.3%), especially those reported from the neighboring countries (17.1% in Peru, 12.1% in Chile, 10.4% in Paraguay and 8.1% in Argentina) [8].

Although the questionnaire has been proven to be valid for eczema and rhinitis symptoms [39,40] it is possible that the results were also affected by interpretation problems related to cultural and educational background differences especially in rural areas. However, the prevalence of rhinitis and eczema symptoms was comparable to previous Latin American studies [4].

Several studies explored the possible relation between ethnicity, poverty, environmental pollution, level of industrialization, health conditions, helminthiasis or genetic factors and the prevalence of asthma and allergies symptoms. The real impact of such factors remains unclear since large differences have been described specially in Latin America [41], but there is common agreement about the importance of environmental factors for the development of asthma and allergic symptoms [42]. Based on the video questionnaire we found a lower prevalence of asthma and allergy symptoms in rural areas where extreme poverty is above 70%, with more than 40% of the population engaged in agricultural activities, more than 90% of the population identifying themselves with native Quechua ethnicity [26] and high percentage of acute respiratory infections and diarrhea in children under 5 years [43].

## Conclusion

Overall in accordance with the hygiene hypothesis, children living in urban areas of Bolivia seem to have a higher prevalence of asthma and atopic symptoms compared to children living in the country side. More research is needed assessing the role of environmental factors in the development of asthma and allergies trying to explain the differences in rural and urban areas. It will help to design future primary prevention strategies in order to avoid the rapid increase of these diseases in this region.

## Competing interests

No conflicts of interests are reported for this study.

## Authors’ contributions

MTSS participated in the design of the study acquisition of data, and performed the statistical analysis. KR conceived of the study, and participated in its design and coordination and helped to draft the manuscript. All authors read and approved the final manuscript.

## Pre-publication history

The pre-publication history for this paper can be accessed here:

http://www.biomedcentral.com/1471-2466/14/40/prepub
